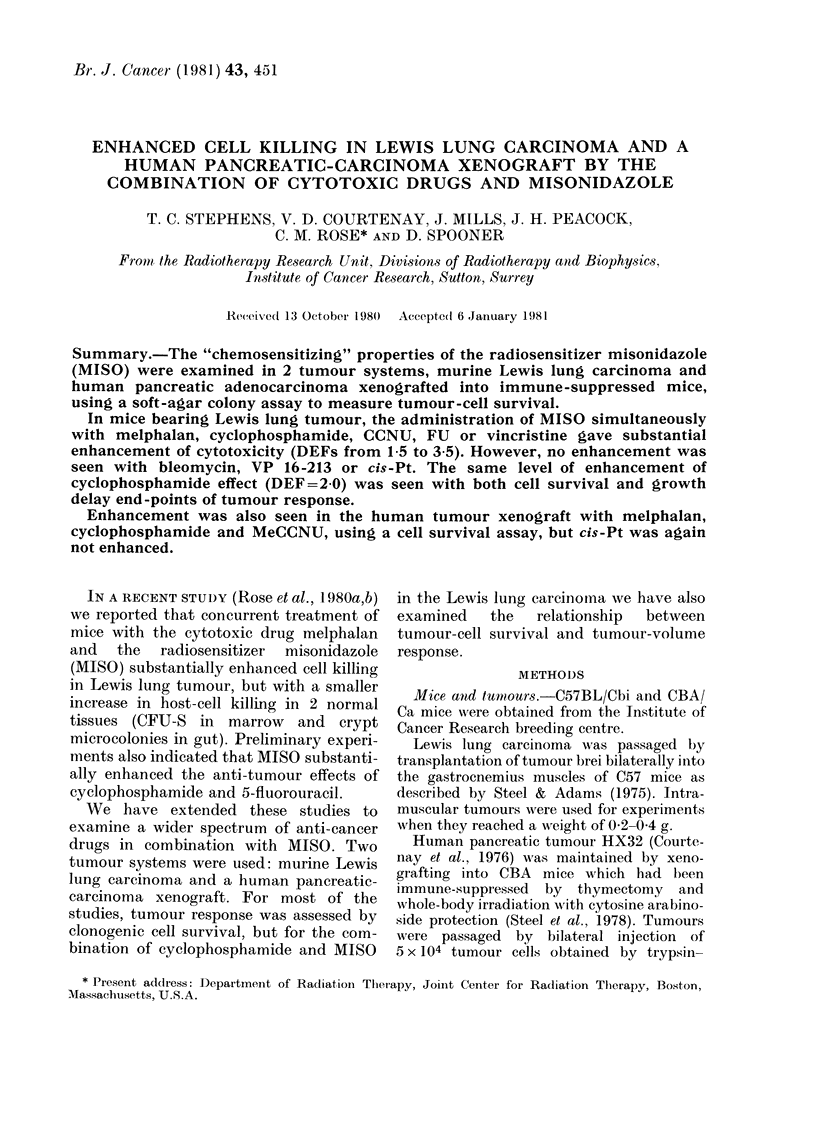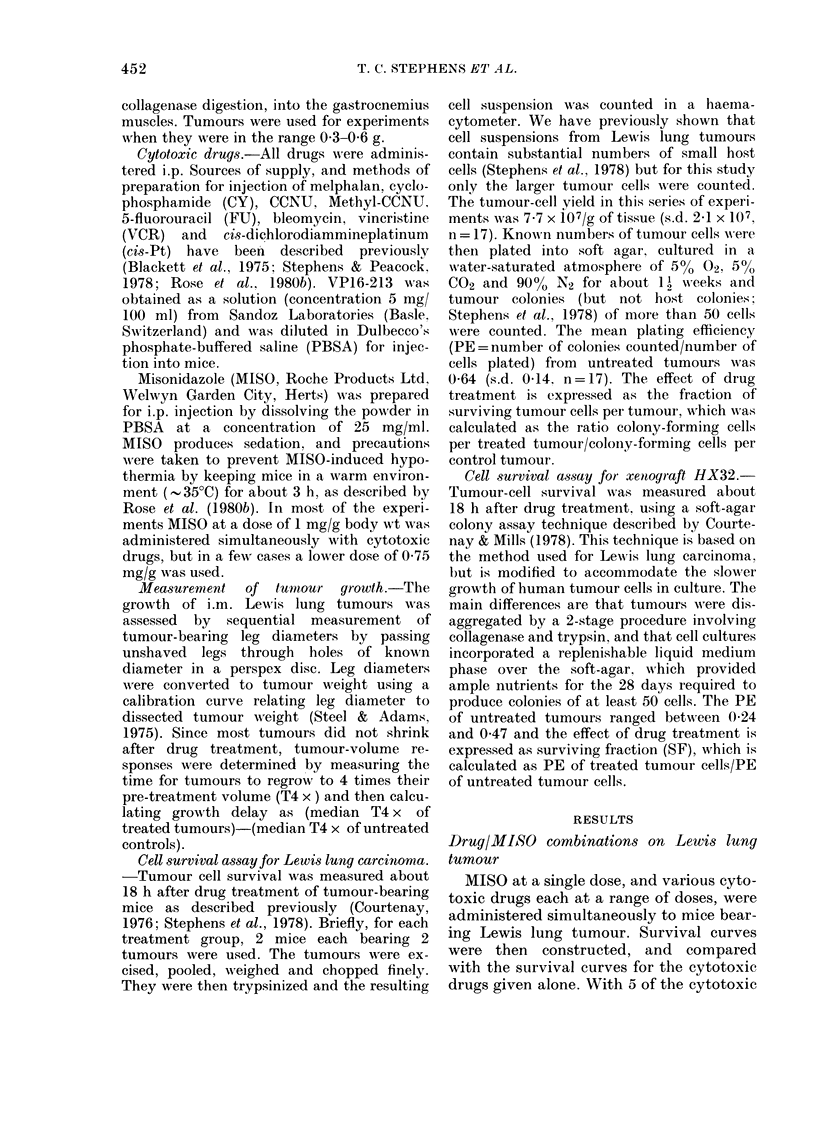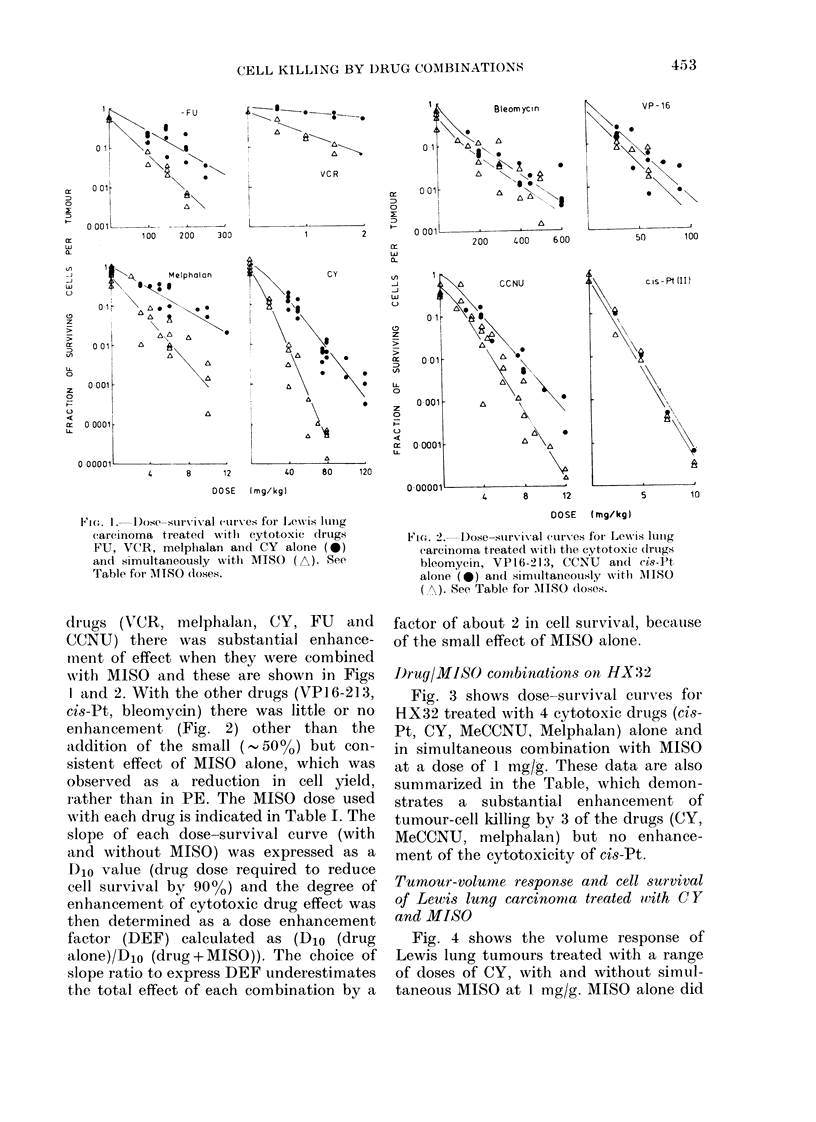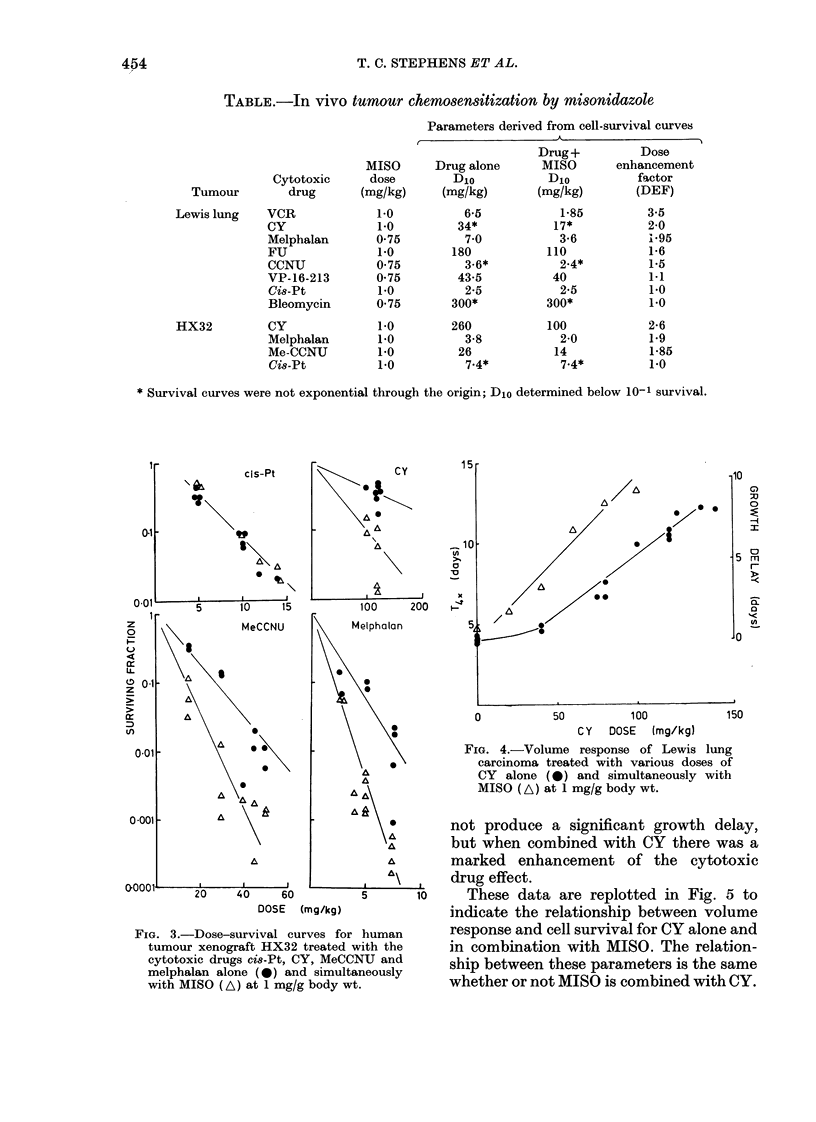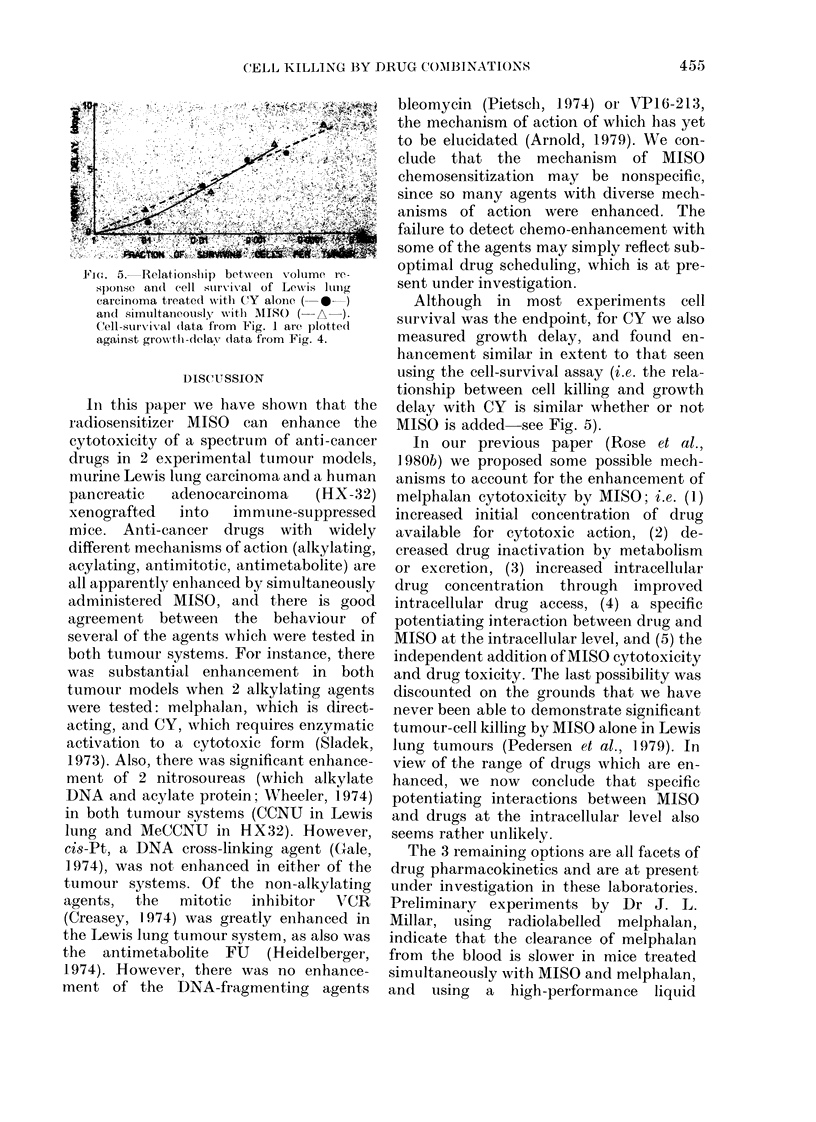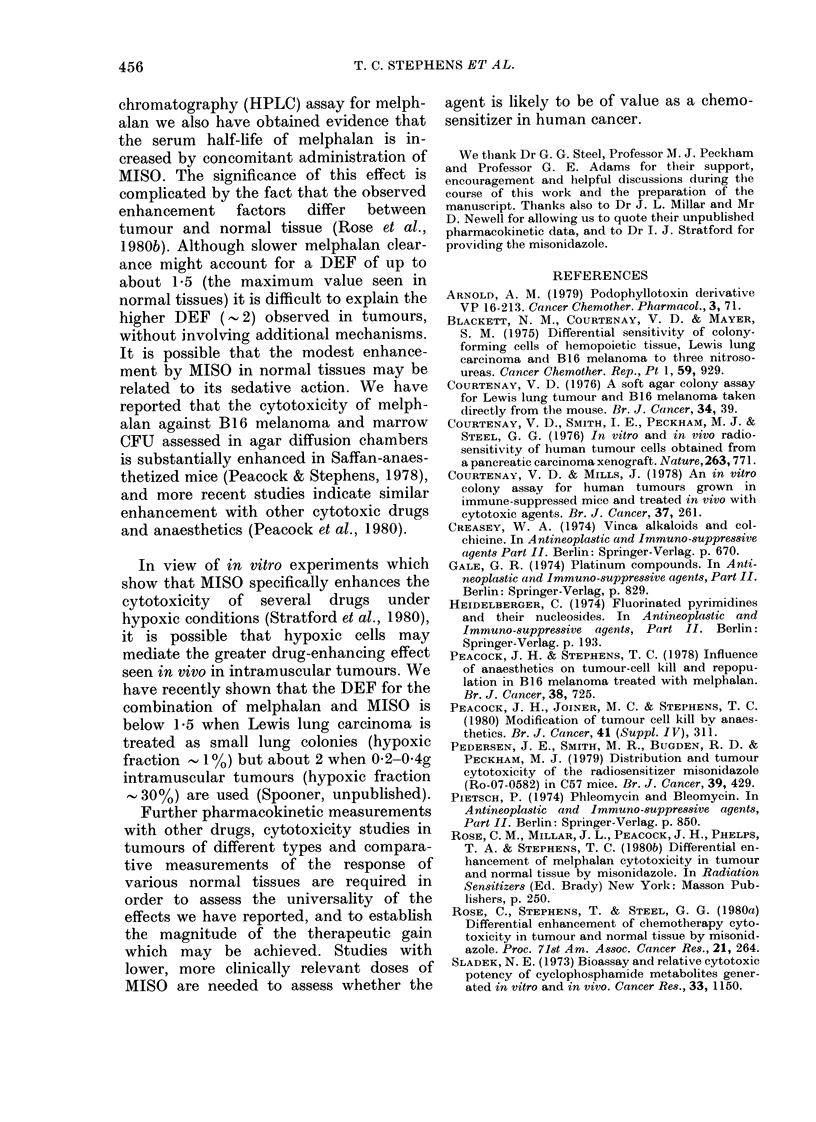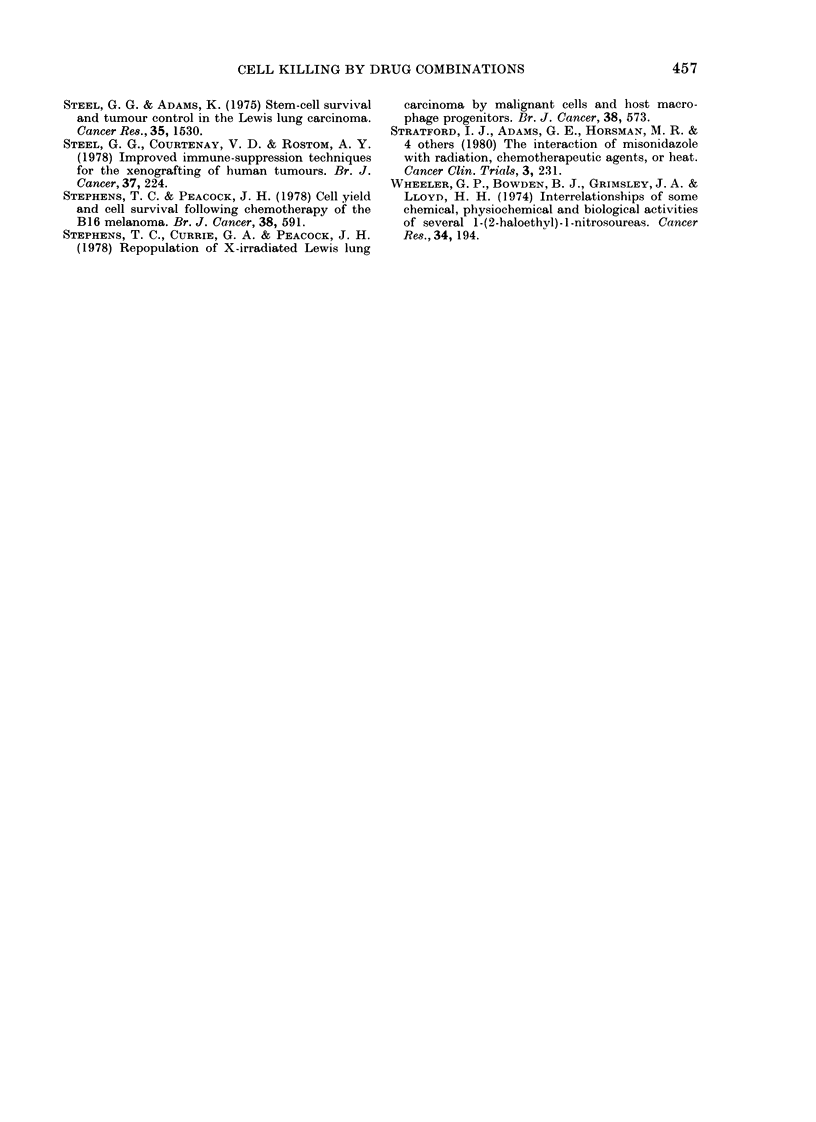# Enhanced cell killing in lewis lung carcinoma and a human pancreatic-carcinoma xenograft by the combination of cytotoxic drugs and misonidazole.

**DOI:** 10.1038/bjc.1981.66

**Published:** 1981-04

**Authors:** T. C. Stephens, V. D. Courtenay, J. Mills, J. H. Peacock, C. M. Rose, D. Spooner

## Abstract

The "chemosensitizing" properties of the radiosensitizer misonidazole (MISO) were examined in 2 tumour systems, murine Lewis lung carcinoma and human pancreatic adenocarcinoma xenografted into immune-suppressed mice, using a soft-agar colony assay to measure tumour-cell survival. In mice bearing Lewis lung tumour, the administration of MISO simultaneously with melphalan, cyclophosphamide. CCNU, FU or vincristine gave substantial enhancement of cytotoxicity (DEFs from 1.5 to 3.5). However, no enhancement was seen with bleomycin, VP 16-213 or cis-Pt. The same level of enhancement of cyclophosphamide effect (DEF = 2.0) was seen with both cell survival and growth delay end-points effect (DEF = 2.0) was seen with both cell survival and growth delay end-points of tumour response. Enhancement was also seen in the human tumour xenograft with melphalan, cyclophosphamide and MeCCNU, using a cell survival assay, but cis-Pt was again not enhanced.


*Br. J. Cancer* (1981) **43**, 451

### ENHANCED CELL KILLING IN LEWIS LUNG CARCINOMA AND A HUMAN PANCREATIC-CARCINOMA XENOGRAFT BY THE COMBINATION OF CYTOTOXIC DRUGS AND MISONIDAZOLE

T. C. STEPHENS, V. D. COURTENAY, J. MILLS, J. H. PEACOCK, C. M. ROSE* AND D. SPOONER

*From the Radiotherapy Research Unit, Divisions of Radiotherapy and Biophysics, Institute of Cancer Research, Sutton, Surrey*

Received 13 October 1980 Accepted 6 January 1981

**Summary.—The "chemosensitizing" properties of the radiosensitizer misonidazole (MISO) were examined in 2 tumour systems, murine Lewis lung carcinoma and human pancreatic adenocarcinoma xenografted into immune-suppressed mice, using a soft-agar colony assay to measure tumour-cell survival.**

**In mice bearing Lewis lung tumour, the administration of MISO simultaneously with melphalan, cyclophosphamide, CCNU, FU or vincristine gave substantial enhancement of cytotoxicity (DEFs from 1·5 to 3·5). However, no enhancement was seen with bleomycin, VP 16-213 or *cis*-Pt. The same level of enhancement of cyclophosphamide effect (DEF=2·0) was seen with both cell survival and growth delay end-points of tumour response.**

**Enhancement was also seen in the human tumour xenograft with melphalan, cyclophosphamide and MeCCNU, using a cell survival assay, but *cis*-Pt was again not enhanced.**

IN A RECENT STUDY (Rose *et al.*, 1980*a*,*b*) we reported that concurrent treatment of mice with the cytotoxic drug melphalan and the radiosensitizer misonidazole (MISO) substantially enhanced cell killing in Lewis lung tumour, but with a smaller increase in host-cell killing in 2 normal tissues (CFU-S in marrow and crypt microcolonies in gut). Preliminary experiments also indicated that MISO substantially enhanced the anti-tumour effects of cyclophosphamide and 5-fluorouracil.

We have extended these studies to examine a wider spectrum of anti-cancer drugs in combination with MISO. Two tumour systems were used: murine Lewis lung carcinoma and a human pancreatic-carcinoma xenograft. For most of the studies, tumour response was assessed by clonogenic cell survival, but for the combination of cyclophosphamide and MISO in the Lewis lung carcinoma we have also examined the relationship between tumour-cell survival and tumour-volume response.

### METHODS

*Mice and tumours.*—C57BL/Cbi and CBA/Ca mice were obtained from the Institute of Cancer Research breeding centre.

Lewis lung carcinoma was passaged by transplantation of tumour brei bilaterally into the gastrocnemius muscles of C57 mice as described by Steel & Adams (1975). Intramuscular tumours were used for experiments when they reached a weight of 0·2–0·4 g.

Human pancreatic tumour HX32 (Courtenay *et al.*, 1976) was maintained by xenografting into CBA mice which had been immune-suppressed by thymectomy and whole-body irradiation with cytosine arabinoside protection (Steel *et al.*, 1978). Tumours were passaged by bilateral injection of $5 \times 10^4$ tumour cells obtained by trypsin–

* Present address: Department of Radiation Therapy, Joint Center for Radiation Therapy, Boston, Massachusetts, U.S.A.

collagenase digestion, into the gastrocnemius muscles. Tumours were used for experiments when they were in the range 0·3–0·6 g.

*Cytotoxic drugs.*—All drugs were administered i.p. Sources of supply, and methods of preparation for injection of melphalan, cyclophosphamide (CY), CCNU, Methyl-CCNU, 5-fluorouracil (FU), bleomycin, vincristine (VCR) and *cis*-dichlorodiammineplatinum (*cis*-Pt) have been described previously (Blackett *et al.*, 1975; Stephens & Peacock, 1978; Rose *et al.*, 1980*b*). VP16-213 was obtained as a solution (concentration 5 mg/100 ml) from Sandoz Laboratories (Basle, Switzerland) and was diluted in Dulbecco's phosphate-buffered saline (PBSA) for injection into mice.

Misonidazole (MISO, Roche Products Ltd, Welwyn Garden City, Herts) was prepared for i.p. injection by dissolving the powder in PBSA at a concentration of 25 mg/ml. MISO produces sedation, and precautions were taken to prevent MISO-induced hypothermia by keeping mice in a warm environment ($\sim$35°C) for about 3 h, as described by Rose *et al.* (1980*b*). In most of the experiments MISO at a dose of 1 mg/g body wt was administered simultaneously with cytotoxic drugs, but in a few cases a lower dose of 0·75 mg/g was used.

*Measurement of tumour growth.*—The growth of i.m. Lewis lung tumours was assessed by sequential measurement of tumour-bearing leg diameters by passing unshaved legs through holes of known diameter in a perspex disc. Leg diameters were converted to tumour weight using a calibration curve relating leg diameter to dissected tumour weight (Steel & Adams, 1975). Since most tumours did not shrink after drug treatment, tumour-volume responses were determined by measuring the time for tumours to regrow to 4 times their pre-treatment volume (T4 $\times$) and then calculating growth delay as (median T4$\times$ of treated tumours)—(median T4 $\times$ of untreated controls).

*Cell survival assay for Lewis lung carcinoma.*—Tumour cell survival was measured about 18 h after drug treatment of tumour-bearing mice as described previously (Courtenay, 1976; Stephens *et al.*, 1978). Briefly, for each treatment group, 2 mice each bearing 2 tumours were used. The tumours were excised, pooled, weighed and chopped finely. They were then trypsinized and the resulting cell suspension was counted in a haemacytometer. We have previously shown that cell suspensions from Lewis lung tumours contain substantial numbers of small host cells (Stephens *et al.*, 1978) but for this study only the larger tumour cells were counted. The tumour-cell yield in this series of experiments was $7{\cdot}7 \times 10^7$/g of tissue (s.d. $2{\cdot}1 \times 10^7$, n = 17). Known numbers of tumour cells were then plated into soft agar, cultured in a water-saturated atmosphere of 5% $O_2$, 5% $CO_2$ and 90% $N_2$ for about $1\frac{1}{2}$ weeks and tumour colonies (but not host colonies; Stephens *et al.*, 1978) of more than 50 cells were counted. The mean plating efficiency (PE = number of colonies counted/number of cells plated) from untreated tumours was 0·64 (s.d. 0·14, n = 17). The effect of drug treatment is expressed as the fraction of surviving tumour cells per tumour, which was calculated as the ratio colony-forming cells per treated tumour/colony-forming cells per control tumour.

*Cell survival assay for xenograft HX32.*—Tumour-cell survival was measured about 18 h after drug treatment, using a soft-agar colony assay technique described by Courtenay & Mills (1978). This technique is based on the method used for Lewis lung carcinoma, but is modified to accommodate the slower growth of human tumour cells in culture. The main differences are that tumours were disaggregated by a 2-stage procedure involving collagenase and trypsin, and that cell cultures incorporated a replenishable liquid medium phase over the soft-agar, which provided ample nutrients for the 28 days required to produce colonies of at least 50 cells. The PE of untreated tumours ranged between 0·24 and 0·47 and the effect of drug treatment is expressed as surviving fraction (SF), which is calculated as PE of treated tumour cells/PE of untreated tumour cells.

### RESULTS

### *Drug/MISO combinations on Lewis lung tumour*

MISO at a single dose, and various cytotoxic drugs each at a range of doses, were administered simultaneously to mice bearing Lewis lung tumour. Survival curves were then constructed, and compared with the survival curves for the cytotoxic drugs given alone. With 5 of the cytotoxic

FIG. 1.—Dose–survival curves for Lewis lung carcinoma treated with cytotoxic drugs FU, VCR, melphalan and CY alone (●) and simultaneously with MISO (△). See Table for MISO doses.

FIG. 2.—Dose–survival curves for Lewis lung carcinoma treated with the cytotoxic drugs bleomycin, VP16-213, CCNU and *cis*-Pt alone (●) and simultaneously with MISO (△). See Table for MISO doses.

drugs (VCR, melphalan, CY, FU and CCNU) there was substantial enhancement of effect when they were combined with MISO and these are shown in Figs 1 and 2. With the other drugs (VP16-213, *cis*-Pt, bleomycin) there was little or no enhancement (Fig. 2) other than the addition of the small ($\sim 50\%$) but consistent effect of MISO alone, which was observed as a reduction in cell yield, rather than in PE. The MISO dose used with each drug is indicated in Table I. The slope of each dose–survival curve (with and without MISO) was expressed as a $D_{10}$ value (drug dose required to reduce cell survival by 90%) and the degree of enhancement of cytotoxic drug effect was then determined as a dose enhancement factor (DEF) calculated as ($D_{10}$ (drug alone)/$D_{10}$ (drug + MISO)). The choice of slope ratio to express DEF underestimates the total effect of each combination by a factor of about 2 in cell survival, because of the small effect of MISO alone.

### *Drug/MISO combinations on HX32*

Fig. 3 shows dose–survival curves for HX32 treated with 4 cytotoxic drugs (*cis*-Pt, CY, MeCCNU, Melphalan) alone and in simultaneous combination with MISO at a dose of 1 mg/g. These data are also summarized in the Table, which demonstrates a substantial enhancement of tumour-cell killing by 3 of the drugs (CY, MeCCNU, melphalan) but no enhancement of the cytotoxicity of *cis*-Pt.

### *Tumour-volume response and cell survival of Lewis lung carcinoma treated with CY and MISO*

Fig. 4 shows the volume response of Lewis lung tumours treated with a range of doses of CY, with and without simultaneous MISO at 1 mg/g. MISO alone did

TABLE.—In vivo *tumour chemosensitization by misonidazole*

| Tumour | Cytotoxic drug | MISO dose (mg/kg) | Parameters derived from cell-survival curves: Drug alone $D_{10}$ (mg/kg) | Drug + MISO $D_{10}$ (mg/kg) | Dose enhancement factor (DEF) |
|---|---|---|---|---|---|
| Lewis lung | VCR | 1·0 | 6·5 | 1·85 | 3·5 |
| | CY | 1·0 | 34* | 17* | 2·0 |
| | Melphalan | 0·75 | 7·0 | 3·6 | 1·95 |
| | FU | 1·0 | 180 | 110 | 1·6 |
| | CCNU | 0·75 | 3·6* | 2·4* | 1·5 |
| | VP-16-213 | 0·75 | 43·5 | 40 | 1·1 |
| | *Cis*-Pt | 1·0 | 2·5 | 2·5 | 1·0 |
| | Bleomycin | 0·75 | 300* | 300* | 1·0 |
| HX32 | CY | 1·0 | 260 | 100 | 2·6 |
| | Melphalan | 1·0 | 3·8 | 2·0 | 1·9 |
| | Me-CCNU | 1·0 | 26 | 14 | 1·85 |
| | *Cis*-Pt | 1·0 | 7·4* | 7·4* | 1·0 |

* Survival curves were not exponential through the origin; $D_{10}$ determined below $10^{-1}$ survival.

FIG. 3.—Dose-survival curves for human tumour xenograft HX32 treated with the cytotoxic drugs *cis*-Pt, CY, MeCCNU and melphalan alone (●) and simultaneously with MISO (△) at 1 mg/g body wt.

FIG. 4.—Volume response of Lewis lung carcinoma treated with various doses of CY alone (●) and simultaneously with MISO (△) at 1 mg/g body wt.

not produce a significant growth delay, but when combined with CY there was a marked enhancement of the cytotoxic drug effect.

These data are replotted in Fig. 5 to indicate the relationship between volume response and cell survival for CY alone and in combination with MISO. The relationship between these parameters is the same whether or not MISO is combined with CY.

FIG. 5.—Relationship between volume response and cell survival of Lewis lung carcinoma treated with CY alone (—●—) and simultaneously with MISO (—△—). Cell-survival data from Fig. 1 are plotted against growth-delay data from Fig. 4.

### DISCUSSION

In this paper we have shown that the radiosensitizer MISO can enhance the cytotoxicity of a spectrum of anti-cancer drugs in 2 experimental tumour models, murine Lewis lung carcinoma and a human pancreatic adenocarcinoma (HX-32) xenografted into immune-suppressed mice. Anti-cancer drugs with widely different mechanisms of action (alkylating, acylating, antimitotic, antimetabolite) are all apparently enhanced by simultaneously administered MISO, and there is good agreement between the behaviour of several of the agents which were tested in both tumour systems. For instance, there was substantial enhancement in both tumour models when 2 alkylating agents were tested: melphalan, which is direct-acting, and CY, which requires enzymatic activation to a cytotoxic form (Sladek, 1973). Also, there was significant enhancement of 2 nitrosoureas (which alkylate DNA and acylate protein; Wheeler, 1974) in both tumour systems (CCNU in Lewis lung and MeCCNU in HX32). However, *cis*-Pt, a DNA cross-linking agent (Gale, 1974), was not enhanced in either of the tumour systems. Of the non-alkylating agents, the mitotic inhibitor VCR (Creasey, 1974) was greatly enhanced in the Lewis lung tumour system, as also was the antimetabolite FU (Heidelberger, 1974). However, there was no enhancement of the DNA-fragmenting agents bleomycin (Pietsch, 1974) or VP16-213, the mechanism of action of which has yet to be elucidated (Arnold, 1979). We conclude that the mechanism of MISO chemosensitization may be nonspecific, since so many agents with diverse mechanisms of action were enhanced. The failure to detect chemo-enhancement with some of the agents may simply reflect sub-optimal drug scheduling, which is at present under investigation.

Although in most experiments cell survival was the endpoint, for CY we also measured growth delay, and found enhancement similar in extent to that seen using the cell-survival assay (*i.e.* the relationship between cell killing and growth delay with CY is similar whether or not MISO is added—see Fig. 5).

In our previous paper (Rose *et al.*, 1980*b*) we proposed some possible mechanisms to account for the enhancement of melphalan cytotoxicity by MISO; *i.e.* (1) increased initial concentration of drug available for cytotoxic action, (2) decreased drug inactivation by metabolism or excretion, (3) increased intracellular drug concentration through improved intracellular drug access, (4) a specific potentiating interaction between drug and MISO at the intracellular level, and (5) the independent addition of MISO cytotoxicity and drug toxicity. The last possibility was discounted on the grounds that we have never been able to demonstrate significant tumour-cell killing by MISO alone in Lewis lung tumours (Pedersen *et al.*, 1979). In view of the range of drugs which are enhanced, we now conclude that specific potentiating interactions between MISO and drugs at the intracellular level also seems rather unlikely.

The 3 remaining options are all facets of drug pharmacokinetics and are at present under investigation in these laboratories. Preliminary experiments by Dr J. L. Millar, using radiolabelled melphalan, indicate that the clearance of melphalan from the blood is slower in mice treated simultaneously with MISO and melphalan, and using a high-performance liquid chromatography (HPLC) assay for melphalan we also have obtained evidence that the serum half-life of melphalan is increased by concomitant administration of MISO. The significance of this effect is complicated by the fact that the observed enhancement factors differ between tumour and normal tissue (Rose *et al.*, 1980*b*). Although slower melphalan clearance might account for a DEF of up to about 1·5 (the maximum value seen in normal tissues) it is difficult to explain the higher DEF ($\sim$2) observed in tumours, without involving additional mechanisms. It is possible that the modest enhancement by MISO in normal tissues may be related to its sedative action. We have reported that the cytotoxicity of melphalan against B16 melanoma and marrow CFU assessed in agar diffusion chambers is substantially enhanced in Saffan-anaesthetized mice (Peacock & Stephens, 1978), and more recent studies indicate similar enhancement with other cytotoxic drugs and anaesthetics (Peacock *et al.*, 1980).

In view of *in vitro* experiments which show that MISO specifically enhances the cytotoxicity of several drugs under hypoxic conditions (Stratford *et al.*, 1980), it is possible that hypoxic cells may mediate the greater drug-enhancing effect seen *in vivo* in intramuscular tumours. We have recently shown that the DEF for the combination of melphalan and MISO is below 1·5 when Lewis lung carcinoma is treated as small lung colonies (hypoxic fraction $\sim$1%) but about 2 when 0·2–0·4g intramuscular tumours (hypoxic fraction $\sim$30%) are used (Spooner, unpublished).

Further pharmacokinetic measurements with other drugs, cytotoxicity studies in tumours of different types and comparative measurements of the response of various normal tissues are required in order to assess the universality of the effects we have reported, and to establish the magnitude of the therapeutic gain which may be achieved. Studies with lower, more clinically relevant doses of MISO are needed to assess whether the agent is likely to be of value as a chemosensitizer in human cancer.

We thank Dr G. G. Steel, Professor M. J. Peckham and Professor G. E. Adams for their support, encouragement and helpful discussions during the course of this work and the preparation of the manuscript. Thanks also to Dr J. L. Millar and Mr D. Newell for allowing us to quote their unpublished pharmacokinetic data, and to Dr I. J. Stratford for providing the misonidazole.

### REFERENCES

ARNOLD, A. M. (1979) Podophyllotoxin derivative VP 16-213. *Cancer Chemother. Pharmacol.*, **3**, 71.

BLACKETT, N. M., COURTENAY, V. D. & MAYER, S. M. (1975) Differential sensitivity of colony-forming cells of hemopoietic tissue, Lewis lung carcinoma and B16 melanoma to three nitrosoureas. *Cancer Chemother. Rep., Pt* 1, **59**, 929.

COURTENAY, V. D. (1976) A soft agar colony assay for Lewis lung tumour and B16 melanoma taken directly from the mouse. *Br. J. Cancer*, **34**, 39.

COURTENAY, V. D., SMITH, I. E., PECKHAM, M. J. & STEEL, G. G. (1976) *In vitro* and *in vivo* radiosensitivity of human tumour cells obtained from a pancreatic carcinoma xenograft. *Nature*, **263**, 771.

COURTENAY, V. D. & MILLS, J. (1978) An *in vitro* colony assay for human tumours grown in immune-suppressed mice and treated *in vivo* with cytotoxic agents. *Br. J. Cancer*, **37**, 261.

CREASEY, W. A. (1974) Vinca alkaloids and colchicine. In *Antineoplastic and Immuno-suppressive agents Part II*. Berlin: Springer-Verlag. p. 670.

GALE, G. R. (1974) Platinum compounds. In *Antineoplastic and Immuno-suppressive agents, Part II*. Berlin: Springer-Verlag, p. 829.

HEIDELBERGER, C. (1974) Fluorinated pyrimidines and their nucleosides. In *Antineoplastic and Immuno-suppressive agents, Part II*. Berlin: Springer-Verlag. p. 193.

PEACOCK, J. H. & STEPHENS, T. C. (1978) Influence of anaesthetics on tumour-cell kill and repopulation in B16 melanoma treated with melphalan. *Br. J. Cancer*, **38**, 725.

PEACOCK, J. H., JOINER, M. C. & STEPHENS, T. C. (1980) Modification of tumour cell kill by anaesthetics. *Br. J. Cancer*, **41** (*Suppl. IV*), 311.

PEDERSEN, J. E., SMITH, M. R., BUGDEN, R. D. & PECKHAM, M. J. (1979) Distribution and tumour cytotoxicity of the radiosensitizer misonidazole (Ro-07-0582) in C57 mice. *Br. J. Cancer*, **39**, 429.

PIETSCH, P. (1974) Phleomycin and Bleomycin. In *Antineoplastic and Immuno-suppressive agents, Part II*. Berlin: Springer-Verlag. p. 850.

ROSE, C. M., MILLAR, J. L., PEACOCK, J. H., PHELPS, T. A. & STEPHENS, T. C. (1980*b*) Differential enhancement of melphalan cytotoxicity in tumour and normal tissue by misonidazole. In *Radiation Sensitizers* (Ed. Brady) New York: Masson Publishers, p. 250.

ROSE, C., STEPHENS, T. & STEEL, G. G. (1980*a*) Differential enhancement of chemotherapy cytotoxicity in tumour and normal tissue by misonidazole. *Proc. 71st Am. Assoc. Cancer Res.*, **21**, 264.

SLADEK, N. E. (1973) Bioassay and relative cytotoxic potency of cyclophosphamide metabolites generated *in vitro* and *in vivo*. *Cancer Res.*, **33**, 1150.

Steel, G. G. & Adams, K. (1975) Stem-cell survival and tumour control in the Lewis lung carcinoma. *Cancer Res.*, **35**, 1530.

Steel, G. G., Courtenay, V. D. & Rostom, A. Y. (1978) Improved immune-suppression techniques for the xenografting of human tumours. *Br. J. Cancer*, **37**, 224.

Stephens, T. C. & Peacock, J. H. (1978) Cell yield and cell survival following chemotherapy of the B16 melanoma. *Br. J. Cancer*, **38**, 591.

Stephens, T. C., Currie, G. A. & Peacock, J. H. (1978) Repopulation of X-irradiated Lewis lung carcinoma by malignant cells and host macrophage progenitors. *Br. J. Cancer*, **38**, 573.

Stratford, I. J., Adams, G. E., Horsman, M. R. & 4 others (1980) The interaction of misonidazole with radiation, chemotherapeutic agents, or heat. *Cancer Clin. Trials*, **3**, 231.

Wheeler, G. P., Bowden, B. J., Grimsley, J. A. & Lloyd, H. H. (1974) Interrelationships of some chemical, physiochemical and biological activities of several 1-(2-haloethyl)-1-nitrosoureas. *Cancer Res.*, **34**, 194.